# Role of hepatitis B virus X protein in regulating LIM and SH3 protein 1 (LASP-1) expression to mediate proliferation and migration of hepatoma cells

**DOI:** 10.1186/1743-422X-9-163

**Published:** 2012-08-16

**Authors:** Renxian Tang, Fanyun Kong, Lina Hu, Hongjuan You, Peng Zhang, Weidong Du, Kuiyang Zheng

**Affiliations:** 1 Department of Pathogenic biology and Laboratory of Infection and Immunology, Xuzhou Medical College, 84 West Huaihai Road, Xuzhou, 221002, Jiangsu Province, China; 2 Sektion Experimentelle Anaesthesiologie,Universitaetsklinikum Ulm, Ulm, 89075, Germany

**Keywords:** HBx, LASP-1, Hepatocellular carcinoma, Proliferation, Migration

## Abstract

**Background:**

Hepatitis B virus X protein (HBx) has been shown to be responsible for the development of hepatocellular carcinoma (HCC) caused by Hepatitis B virus infection. However, its potential effect on the progression of hepatocellular carcinoma remains yet unclear. LIM and SH3 protein 1 (LASP-1), a focal adhesion protein, is expressed in an up-regulation manner in the HCC tissues. LASP-1 plays an important role in the regulation of proliferation and migration of HCC. In this study, we investigated the effect of LASP-1 involved in HBx-related tumor progression.

**Methods:**

LASP-1 levels in the HBx stable transfected HepG2 and Huh-7 cells were detected by RT-PCR and western blot analysis. The cellular localization of LASP-1 was assessed by immunofluorescence analysis. The activity of phosphatidylinositol 3-kinase (PI3-K) pathway was demonstrated by western blot assay. The HBx-expressing cells were transfected with specific small interference RNA (siRNA) against LASP-1. The proliferation and migration ability of cells were evaluated by cell viability assay and plate clone formation assay. The migration ability of cells was detected by transwell assay and wound healing assay.

**Results:**

RT-PCR and western blot analysis indicated the expression of LASP-1 was increased in the stable HBx-expressing cells compared with the control cells. Immunofluorescence study revealed that the distributions of LASP-1 in HepG2-HBX cells were mainly in pseudopods and the cytoplasm while they were mainly localized in the cytoplasm of HepG2-Mock cells. The cellular localizations of LASP-1 in Huh-7-HBX cells were in the perinuclear fractions while they were mainly localized in the cytoplasm of Huh-7-Mock cells. The upregulation of LASP-1 was inhibited after treatment with LY294002, PI3-K pathway inhibitor. Overexpression of LASP-1 in the stable HBx-expressing cells enhanced the proliferation and migration ability of hepatocellular cells. siRNA-mediated LASP-1 knowdown in the stable HBx-expressing cells significantly suppressed hepatocellular cells proliferation and migration.

**Conclusions:**

These results demonstrated that HBx could upregulate LASP-1 through PI3-K pathway to promote the proliferation and migration of hepatoma cells.

## Background

Hepatitis B virus (HBV) infection is one of the most widely spread viral diseases and strongly associated with the development of hepatocellular carcinoma (HCC). However, the mechanism of HBV-mediated HCC development is not yet clearly elucidated. HBx, the protein encoded by the X gene of the HBV genome, is a multifunctional regulatory protein and has been implicated in HBV-mediated hepatocarcinogenesis [1]. Studies have identified that HBx could stimulate HBV replication and interfere with cell cycle progression through interacting with many cellular regulatory proteins including p53, AP-1, the cyclic AMP response element-binding protein (CREB) and activating transcription factor 2 (ATF2) [2]. HBx has been reported to be capable of activating several signal transduction pathways, such as mitogen-activated protein kinase, Ras-Raf-mitogen-activated protein kinase, and JAK/STAT signaling pathways to affect several cellular processes, including proliferation and differentiation [2,3]. Furthermore, HBx has an inhibitory effect on DNA repair that contributes to HBV-induced tumorigenesis [4]. In contrast to its proliferative effects, HBx also participates in inducing cell death by the death receptor pathway or affecting mitochondrial pathophysiological microenvironment to mediate apoptosis [5-8]. Some studies have demonstrated that HBx also contributes to invasion and metastasis of hepatocellular carcinoma by inducing a migratory phenotype in transformed cells in a CD44-dependent manner and altering ECM adhesion properties of HBx-bearing cell by interfering with the expression of the fibronectin receptor, α5β1 [9,10]. Moreover, HBx plays an important role in tumor spreading by enhancing cellular migration through upregulation of MMP-9, MMP-3, MT1-MMP and COX-2 [11-14].

The LIM and SH3 domain protein 1 (LASP-1) was initially identified from a cDNA library from breast cancer metastases tissue and was mapped to human chromosome 17q21 [15,16]. The human LASP-1 gene encodes a membrane-bound protein of 261 amino acids containing one N-terminal LIM domain, followed by two actin-binding sites and a C-terminal src homology SH3 domain [15,17]. The LIM domain of LASP-1 could directly bind to the carboxy-terminal domain (CTD) of CXCR2 and is critical for CXCR2-mediated chemotaxis [18]. The SH3 domain of LASP-1 is involved in protein-protein interactions through binding to proline-rich sequences, specifically with palladin, lipoma preferred partner (LPP), vasodilator stimulated phosphoprotein (VASP) and zyxin. It is reported that LASP-1 localizes within multiple sites of dynamic actin assembly such as focal contacts, focal adhesions, lamellipodia, membrane ruffles, pseudopodia and involves in cell proliferation and migration [17]**.**

Previous investigations showed that LASP-1 was expressed at a low basal level in some of normal human tissues, but was rich in the central nervous system neurons [19]. In metastatic human breast cancer, ovarian cancer, colorectal cancer, malignant childhood medulloblastoma and hepatocellular carcinoma, overexpression of LASP-1 was demonstrated [20-24]. Furthermore, the increased expression of LASP-1 correlated significantly with tumor size and lymph node metastasis [25,26]. *In vitro* studies showed that LASP-1 played an important role in tumor development and metastases. Knock-down of LASP-1 by RNA interference resulted in a strong inhibition of proliferation and migration of cancer cells, such as breast, ovarian and colorectal cancer cell lines [20-22]. In addition, LASP-1 silencing was associated with a reduced binding of the LASP-1 binding partner zyxin to focal contacts [20]. To date, several researches have been carried out to investigate its regulatory mechanisms. Many studies showed that several factors participated in regulating LASP-1 expression. For example, in invasive breast cancer cells, LASP-1 expression was significantly inversely affected by prostate-derived ETS factor (PDEF), a transcription factor known to repress a variety of genes that are possibly involved in tumorigenesis [27]. In hepatocellular carcinoma, LASP-1 was repressed by wild-type p53 at the transcriptional level. Functional negative p53 mutations led to increased LASP-1 expression [24]. In addition, urokinase-type plasminogen activator (uPA) plays a role in controlling the level of LASP-1 expression in that uPA ectopic up-regulation leads to LASP-1 overexpression [28].

In this study, we investigated the effect of HBx on the regulation of LASP-1. Our findings showed that HBx was able to upregulate the expression of LASP-1 in human hepatoma HepG2 and Huh-7 cells through activation of phosphatidylinositol 3-kinase (PI3-K) pathway. In addition, the upregulation of LASP-1 mediated by HBx contributed to proliferation and migration of hepatoma cells.

## Results

### The expression of HBx induced morphologic changes of hepatoma cells and resulted in more multinucleate cells

**Figure 1:**
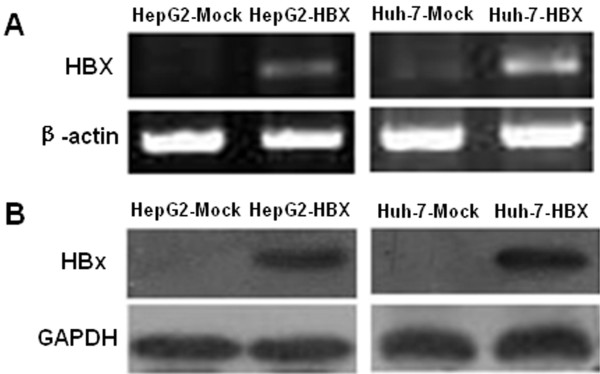
**Detection of HBX expression in stable transfected HepG2 cells and Huh-7 cells.** (**A**): RT-PCR detection of HBX mRNA expression; (**B**): Western blot detection of HBX protein expression.

**Figure 2:**
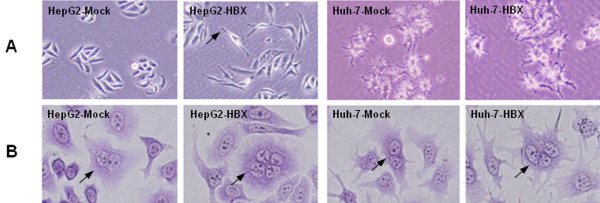
**The morphology and phenotype of control cells and HBx stable expressing cells.** (**A**). The morphology of cultured cells was observed under an inverted microscope (magnification, × 200). The arrow shows the long pseudopod in HepG2-HBX cells. (**B**). The cells were stained with Wright's stain and observed under an inverted microscope (magnification, × 400). The arrow shows HepG2-Mock and Huh-7-Mock cells displayed with double nuclear. In HepG2-HBX and Huh-7-HBX cells, more multinucleate cells could be seen as the arrow shown.

To investigate the potential ability of HBx in regulating LASP-1 expression, we transfected HBx-expressing plasmid pcDNA3.1-X into two human hepatocarcinoma cell lines, HepG2 cells and Huh-7 cells, and established two stable HBx-expressing cell lines, HepG2-HBX and Huh-7-HBX. HepG2 cells and Huh7 cells transfected with empty pcDNA3.1 vector were used as the control cells named HepG2-Mock and Huh-7-Mock. RT-PCR and western blot analysis demonstrated that, compared with the control cells, HBX mRNA and protein were expressed in the HBX stable transfected cells (Figure 1). Interestingly, compared with HepG2-Mock cells, the expression of HBx caused cellular morphological changes indicated by inverted microscopy, which displayed with long pseudopods in the HepG2-HBX cells. This observation indicated that HBx might induce HepG2 cells to display a higher migration capacity (Figure 2A). Compared with the two nucleate cells of HepG2-Mock and Huh-7-Mock stained with Wright's stain, more multinucleate cells of HepG2-HBX and Huh-7-HBX could be observed. The results indicated that HBx could modify the phenotype and confer the migration ability of hepatocarcinoma cells (Figure 2B).

### HBx upregulated expression of LASP-1 in hepatoma cells

**Figure 3:**
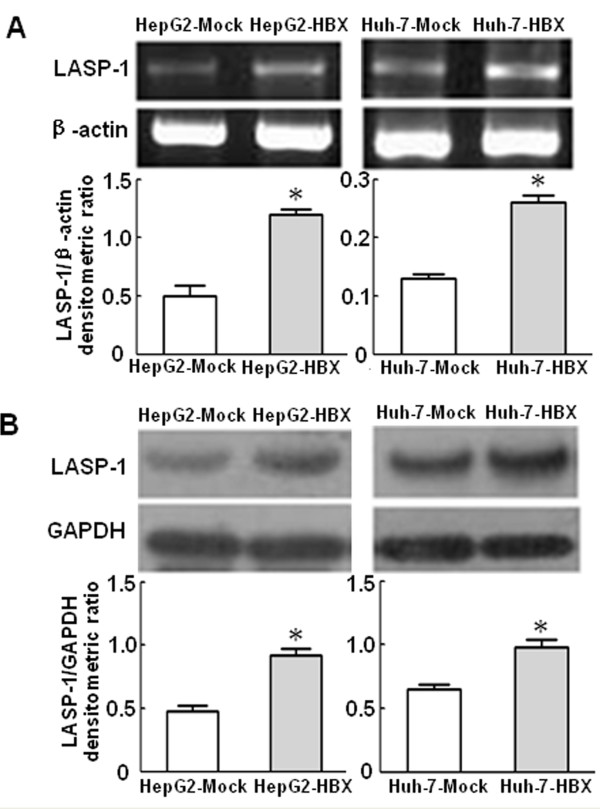
**HBx induced the upregulation of LASP-1.** (**A**): LASP-1 mRNA was detected by RT-RCR. (**B**): LASP-1 protein was detected by western blot analysis. HepG2-Mock cells and Huh-7-Mock cells served as control cells. *P < 0.05 compared with control cells.

**Figure 4:**
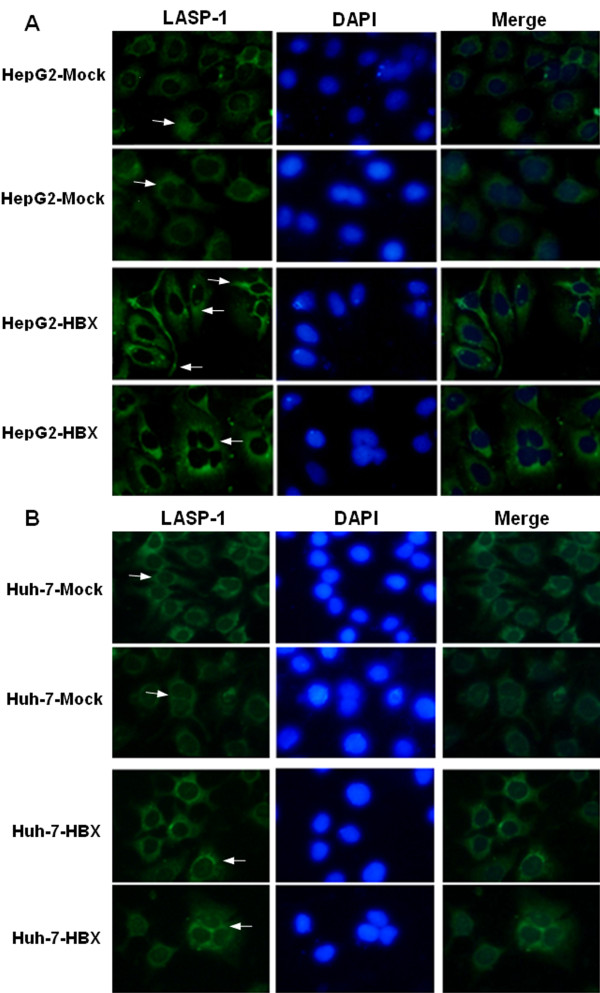
**Fluorescence analysis of Subcellular localization of LASP-1 in cultured cells.** The cells stained with antibodies against LASP-1 (green) and DAPI (blue), the arrow shows that LASP-1 mainly localized in the cytoplasm in normal and double nuclear control cells (**A**, **B**). In stable HBx-expressing cells LASP-1 mainly localized in cytoplasm and pseudopods of normal HepG2-HBX cells displayed with long pseudopods (**A**). In normal Huh-7-HBX cells , LASP-1 predominantly present in cytoplasm (**B**). In multinucleate cells of HepG2-HBX and Huh-7-HBX, LASP-1 mainly localized in the perinuclear (**A**, **B**).

We examined the expression of LASP-1 at the mRNA and protein levels in the control cells and the stable HBx-expressing cells. The RT-PCR and western blot analysis showed that, compared with the control cells, HBx could upregulate the expression of LASP-1 in the stable HBx-expressing cells (Figure 3). Immunofluorescence analysis displayed that the distribution patterns of LASP-1 quite differed in the two cell lines. LASP-1 was mainly localized in pseudopods and the cytoplasm in HepG2-HBX cells and in cytoplasm of HepG2-Mock cells. LASP-1 was localized in the perinuclear fractions in Huh-7-HBX cells as well as in the cytoplasm of Huh-7-Mock cells (Figure 4).We didn’t observe any obvious intranuclear stainings for LASP1 in the control cells and the stable HBx-expressing cells. These date suggested that HBx could influence the subcellular localization of LASP-1 in cytoplasm of human hepatocarcinoma cells.

### HBx-mediated upregulation of LASP-1 requires PI3-K activity

**Figure 5:**
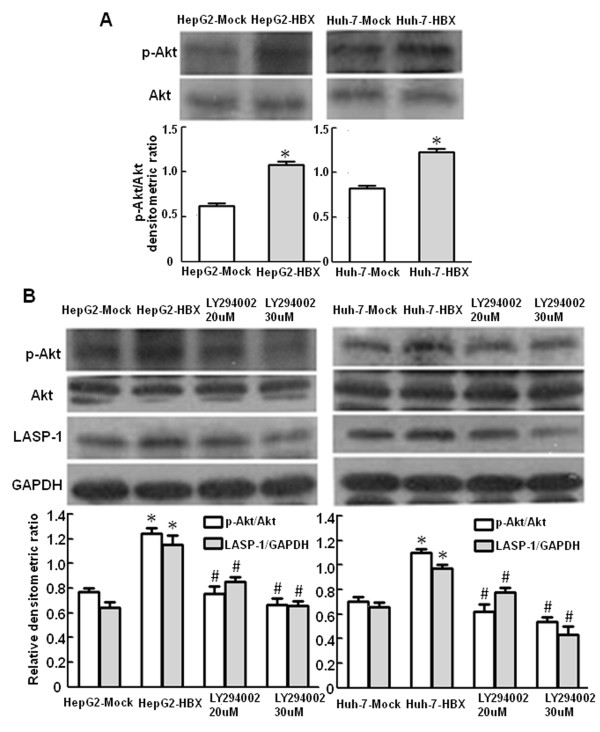
**Activation of PI3-kinase pathway on LASP-1 expression by HBx.** (**A**). HBx induces activation of PI3-kinase in HepG2-HBX and Huh-7-HBX cells. The protein levels of phosphorylated Akt and Akt in cellular extracts were detected by Western blot analysis. HepG2-Mock cells and Huh-7-Mock cells served as controls. (**B**). Activation of PI3-kinase on LASP-1 expression by HBx was inhibited by LY294002. HepG2-HBX and Huh-7-HBX cells were cultured in 0.01% DMSO in the absence or presence of 20 μM or 30uM LY294002 and incubated for 8 h, cell lysates were prepared and subsequently performed by Western blot analysis. *P < 0.05 compared with control cells. ^#^*P* < 0.05 compared with the HBx stable expressing cells.

A recent report showed that LASP-1 expression induced by insulin-like growth factor-I (IGF-I) required activation of PI3-K pathway [29]. To answer whether PI3-K pathway is involved in the regulation of LASP-1mediated by HBx, we detected the phosphorylation level of Akt, a downstream protein of PI3-K pathway in the stable HBx-expressing cells by western blot analysis. The data showed that the phosphorylation level of Akt protein was higher in the stable HBx-expressing cells than the control cells (Figure 5A). Then we treated the stable HBx-expressing cells with the specific PI3-K inhibitor, LY294002. As shown by the results, LY294002 suppressed Akt phosphorylation and LASP-1 expression in a dose dependent manner (Figure 5B). Taken together, these results indicated that the induction of LASP-1 expression by HBx might be regulated by elevated activities of PI3-K pathway.

### HBx promoted the proliferation and migration ability of hepatoma cells through upregulation of LASP-1

**Figure 6:**
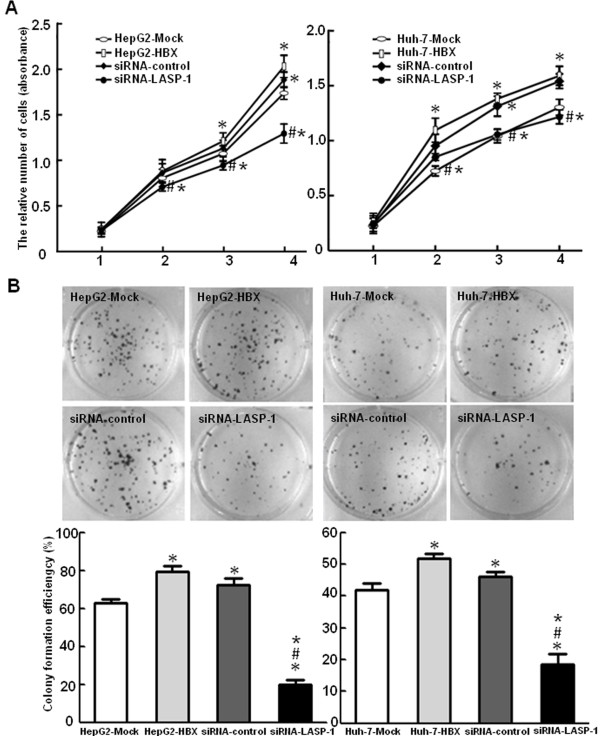
**HBx promotes proliferation ability of HepG2 and Huh-7 cells through upregulation of LASP-1.** (**A**). After transfection with siRNA targeting LASP-1 and incubation for 24 h, the cell proliferation was examined by Cell viability assay. Data were presented as mean ± SD of the absorbance value of cells. (**B**) HepG2-HBx and Huh-7-HBX cells were treated with siRNA targeting LASP-1 for 24 h. Colony formation efficiency was detected by plate clone formation assay. After incubation for two weeks, the cells were stained with Crystal Violet Staining Solution and observed using Bio-Rad Gel Doc XR documentation system. HepG2-Mock and Huh-7-Mock groups served as control groups. HepG2-HBX and Huh-7-HBX groups were HBX stable expressing cells. siRNA-control group was HepG2-HBX or Huh-7-HBX cell transfected with pcDNA™6.2-GW/EmGFP-miR vector as siRNA negative control cell. siRNA-LASP-1 group was HepG2-HBX or Huh-7-HBX cells transfected with siRNA targeting LASP-1 plasmids. *P < 0.05 compared with control cells. ^#^*P* < 0.05 compared with HBx stable expressing cells. *P < 0.05 compared with HBx stable expressing cells transfected with siRNA negative control plasmids.

**Figure 7:**
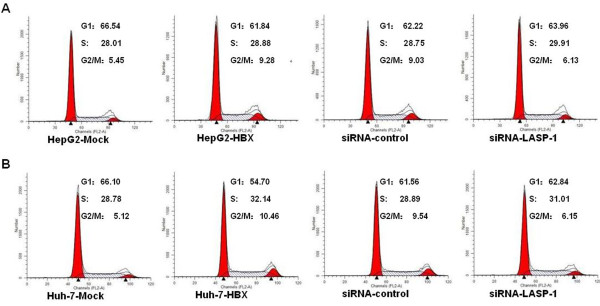
**LASP-1 siRNA treatment decreased the accumulation of HepG2-HBX and Huh-7-HBX cells at G2/M phase of cell cycle.** The groups were described in Figure 6. After transfection with LASP-1 siRNA and incubation for 48 h, the cell cycles were measured with flow cytometry analysis (**A**, **B**).

**Figure 8:**
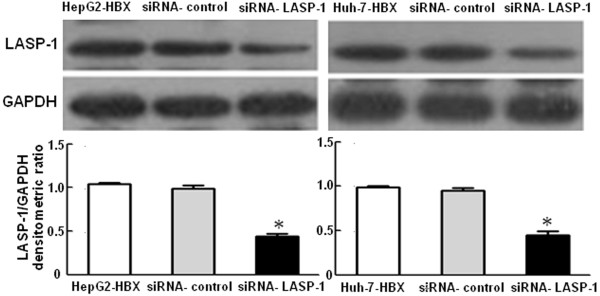
**Effects of siRNA againsting LASP-1 on LASP-1 expression in HepG2-HBX and Huh-7-HBX cells.** Forty-eight hours after transfection, LASP-1 protein expression was determined by western blot analysis. HepG2-HBX group, Huh-7-HBX group and siRNA-control group were used as the negative controls. *P < 0.05 compared with negative controls.

**Figure 9:**
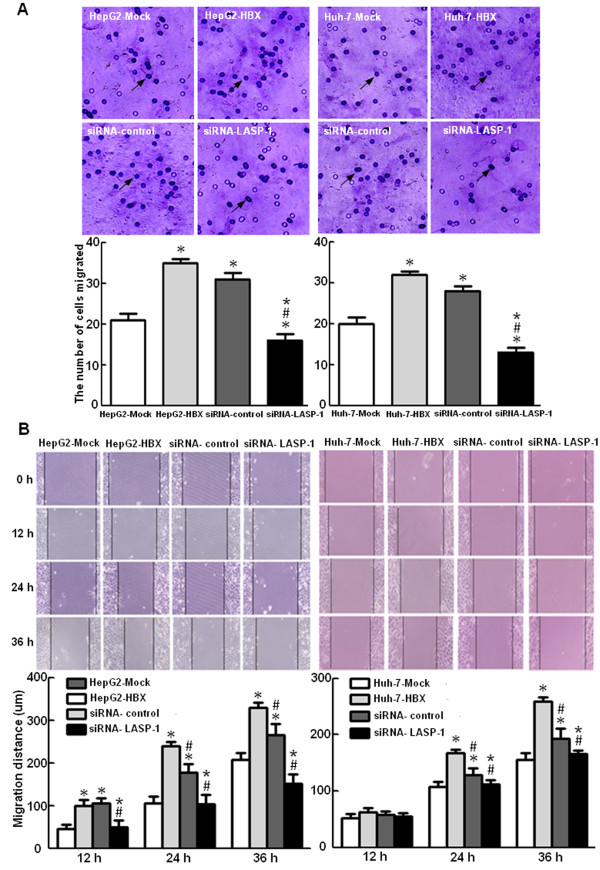
**Incearsed LASP-1 mediated by HBx promotes migration ability of HepG2 and Huh-7 cells.** The groups were described in Figure 6. (**A**). After transfection with siRNA targeting LASP-1 and incubation for 24 h, the cell migration was examined by transwell assay. (**B**). HepG2-HBX and Huh-7-HBX cells were treated with siRNA targeting LASP-1 for 24 h, cell migration ability was examined by wound healing assay. The average migration distances of the wound edge in three independent experiments are quantitated below the photograph data. *P < 0.05 compared with control cells. ^#^*P* < 0.05 compared with stable HBx-expressing cells. *P < 0.05 compared with stable HBx-expressing cells transfected with siRNA negative control plasmids.

To observe the effects of HBx and LASP-1 on cell proliferation, we performed the cell viability assay and plate colony formation assay. Figure 6 displayed that, compared with the control cells, the stable HBx-expressing cells increased proliferation rate and formed more colonies, indicating that HBx could promote the proliferation of hepatocarcinoma cells. To examine whether the upregulation of LASP-1 contributed to proliferation mediated by HBx, we treated the stable HBx-expressing cells with siRNA for LASP-1. As shown in Figure 6, when compared with the stable HBx-expressing cells and the siRNA negative control cells, silencing LASP-1 could significantly suppress proliferation ability of the stable HBx-expressing cells. The cell cycle was also analyzed by flow cytometry. The results showed that a higher percentage of the stable HBx-expressing cells (9.28-10.46%) accumulated in the G2/M phase, compared with that of the control cells (5.12-5.45%). However, when transfected with LASP-1 siRNA, the number of these cells accumulated in G2/M phase was significantly decreased (Figure 7). The siRNA knockdown effect of LASP-1 protein level was detected by Western blot analysis, compared with the negative controls; siRNA could significantly suppress the expression of LASP-1 in the stable HBx-expressing cells (Figure 8). We investigated the relevance of HBx and LASP-1 for cell migration using transwell assay and wound healing assay. The increased cellular migration ability was found in the stable HBx-expressing cells relative to the control cells. Compared to the stable HBx-expressing cells and the siRNA negative control cells, the migratory activity of the stable HBx-expressing cells was inhibited by silencing of LASP-1 (Figure 9). Taken together, these results suggested that HBx was indispensable for enhancing cell proliferation and migration ability by over-expressing LASP-1.

## Discussion

Although chronic HBV infection is responsible for the development of HCC, the specific role of HBx in HCC progression remains unclear. In order to identify the function of HBx in tumorigenesis, we established stable HBx-expressing cell lines and found that HBx could promote aggressive phenotypes of hepatoma cells, which displayed with long pseudopods in HepG2 cells, similar to the previous reports that HBx could induce a migratory phenotype in transformed cells [9,10]. Moreover, we also noticed that HBx induced more multinucleated cells, which might be explained by a recent study that HBx could induce amplification of centrosomes, multipolar spindle formation, and chromosomal missegregation during mitosis through the activation of Ras-extracellular-signal-regulated kinase (MEK)-Mitogen-Activated Protein Pathway (MAPK), and subsequently increase the generation of multinucleated cells [30]. These changes triggered by HBx might confer the acquisition of metastatic properties and genomic instability that contribute to the development of HCC.

In this study, we investigated HBx effect on the expression of LASP-1 in HepG2 and Huh-7 cell lines in order to understand its possible relationship to the HBx-induced HCC. For this purpose, we first analyzed the expression of LASP-1 in the two cell lines with or without HBX gene expression. Our finding showed that the expression of LASP-1 was upregulated in the stably HBX-transfected HepG2 and Huh-7 cell lines by RT-PCR and western blot analysis. We further detected the distribution pattern of LASP-1 in the stable HBx-expressing cells and the control cells. Immunofluorescence study revealed that HBx could influence the cellular distribution of LASP-1 in normal HepG2-HBX and Huh-7-HBX as well as in the multinucleate cells induced by HBx.

The molecular mechanisms involved in the regulation of LASP-1 expression have been explored, but the results were quite controversial. Wang et al. indicated that the transcription of LASP-1 gene was controlled by tumor suppressor gene p53, and inactivation of p53 by mutation would induce the overpression of LASP-1 in hepatoma cells [24]. But Frietsch et al. argued that LASP-1 overexpression was not associated with p53 mutations in breast cancer [25]. In invasive breast cancer cells, the potential tumor suppressor PDEF was identified to repress LASP-1 expression [27]; however inverse correlation between LASP-1 and PDEF levels could not be demonstrated [25]. Welch et al. showed that LASP-1 gene expression could be repressed by transcription factor GATA-1 in murine embryonic stem cells (G1E-ER4 cells)[31]. The various conclusions might be explained by that the LASP-1 gene expression could be regulated by different molecular mechanisms in cancer cell lines and stem cells [20-22]. A recent report revealed that IGF-I-induced LASP-1 expression required activation of PI3-K pathway [29]. Previous investigations indicated that HBx activated PI3-K pathway to suppress the activation of caspase 3 and downregulated TGF-β-induced apoptosis [32,33]. In addition, HBx induced matrix metalloproteinase-9 gene expression that contributed to increase the invasive potential through activation of PI3-K pathway [11]. Based on the investigations described above, in this study, we explored molecular mechanisms of LASP-1 expression induced by HBx. We speculated that PI3-K pathway may be involved in the HBx-mediated upregulation of LASP-1 in hepatoma cells. As expected, our data revealed that HBx increased phosphorylation of Akt, suggesting that HBx could induce activation of PI3-K pathway. In addition, the expression level of LASP-1 was significantly decreased in the HBx-expressing hepatoma cells after the treatment with LY294002, suggesting that PI3-K pathway is responsible for the upregulation of LASP-1 mediated by HBx in the hepatoma cells. PI3-K is a heterodimeric complex that contains two subunits, one is an 85-kDa regulatory protein, p85, and the other is a 110-kDa catalytic protein, p110 [34]. Current researches showed that HBx could mediate PI3-K pathway activation through several molecular mechanisms. For example, HBx has been shown to activate Src family kinases [35]. The SH3 domain of Src family kinases holds function of interacting with a proline-rich region within the p85 to activate PI3-K [36]. HBx was also capable of activating Ras-GTP complex formation [37]. The activated Ras-GTP complex binds to p110, leading to the activation of PI3-K [38]. Besides, Lee YH et al. indicated that HBx could activate Jak-STAT pathway through the interaction with Jak1 to induce the tyrosine phosphorylation of STAT-3 and STAT-5 [39], activated STAT-3 could bind to p85 of PI3-K and activate the pathway [40]. Subsequently, the activated PI3-K pathway regulates several downstream transcription factors, such as NF-kB and CREB, to affect the transcription of multiple genes [41]. The special regulatory elements in the promoter region of LASP-1 gene has not be identified, therefore, further studies should be pursued to define the regulatory elements that control LASP-1 gene transcription in the promoter region and elucidate the precise molecular mechanisms of LASP-1 gene regulation at the transcriptional level by PI3-K signaling pathway.

HBx as a multifunctional viral protein not only promotes cell proliferation but also enhances cell migration. It is reported that HBx could upregulate the activation level of the cyclin-dependent kinase (CDK) 2 and CDC2, and enhance their active association with cyclin E/cyclin A and cyclin B, respectively. It is showed that HBx activates the cyclin A promoter, induce cyclin A–cyclin-dependent kinase 2 complexes and promotes cycling of growth-arrested cells into G1 phase [4]. The ability of HBx to induce tumor cell invasion has been demonstrated by evidences of increasing the expression of VEGF, MMPs, Capn4 and enhancing the capacity of tumor cells to degrade the ECM [10,42,43]. Furthermore, it has been indicated that HBx could induce cell polarization and adhesion receptor redistribution to increase cell migration [9]. In our study, we investigated the role of HBx and LASP-1 in cell proliferation and migration. As shown in Figure 6, the Cell viability assay and plate colony formation assay revealed that HBx enhanced cell proliferation rate and increased the ability to form clones. A higher percentage of the stable HBx-expressing cells in G2/M phase were observed by cell cycle analysis. It was reported that silencing the highly expressed LASP-1 by RNAi significantly influenced the percentage of cells in G2/M phases and reduced cell proliferation [20-22]. In consistence with these findings, the cell proliferation rate and colony formation efficiency were strongly suppressed when transfected with LASP-1 siRNA. The results of cell cycle analyses revealed that LASP-1 siRNA led to a declined percentage of the stable HBx-expressing cells accumulated in G2/M phase. These results indicated that the expression of HBx in the stable HBx-expressing cells affected expression of LASP-1 related to proliferation of HCC.

Next, we observed the effects of HBx and LASP-1 on cell migration. The trasnwell and wound healing assays showed that HBx increased the ability of migration and wound repair in the stable HBx-expressing cells. Meanwhile, the LASP-1 siRNA could abrogate the enhanced migration mediated by HBx. It is known that HBx could induce subcellular redistribution of proteins to the pseudopod and increase their association with CD44 [9]. A previous study indicated that the binding between LASP-1 and Krp1 occurred in co-localization to the membrane-bound integrin CD44 [44]. In this study, we found that increased expression of LASP-1 dominantly located in the pseudopods and the cytoplasm in the HepG2-HBX cells, which implied that LASP-1 might be involved in cells migration mediated by CD44 and this may be partial explain the role of LASP-1 in HBx-mediated migration.

## Conclusion

In summary, our present data demonstrated that HBx upregulated the expression of LASP-1 through PI3-K in promotion of hepatoma cell proliferation and migration, suggesting that LASP-1 might play a critical role in the HBx-associated hepatocarcinogenesis.

## Materials and methods

### Reagents

The RPMI 1640 medium, Dulbecco’s modified Eagle’s medium (DMEM), liposome Lipofectamin 2000, and Trizol reagent were obtained from Invitrogen (Carlsbad, CA, USA). Mouse monoclonal anti-HBx antibody, Mouse monoclonal anti-LASP-1 antibody, Immobilon Western Chemiluminescent HRP Substrate were from millipore (Temecula, CA, USA), rabbit polyclonal anti-phospho-Akt (Ser-473) antibody was from Bioword Technology (Atlanta, GA, USA), rabbit polyclonal anti-Akt antibody, goat anti-mouse IgG-HRP, goat anti-rabbit IgG-HRP were from Santa Cruz (CA, USA), Mouse monoclonal anti-GAPDH antibody, DAPI were from Beyotime Institute of Biotechnology (Jiangsu, China). FITC-conjugated secondary antibody was from Zhongshan Goldenbridge Biotechnology (Beijing, China). Cell counting kit-8 was from Dojindo Laboratories (Kumamoto, Japan). Transwell cluster plates (8.0 μm pore, 24-well) were from Corning Costar (Cambridge, Massachusetts, USA). Primers were synthesized by Shanghai Sangon Biological Engineering Technology and Services (Shanghai, China). TIANScript RT Kit was from TIANGEN Biotech (Beijing, China). G418 was purchased from Promega (Madison, WI, USA), A 50 mg/ml stock solution of G418 was prepared in 100 mM HEPES (pH 7.3) and stored at 4°C. LY294002 was obtained from Sigma (St. Louis, MO, USA). A 100 μM stock solution of LY294002 was prepared in dimethyl sulfoxide (DMSO) and stored at 4°C in the dark. Treatment concentration of LY294002 was prepared fresh for each experiment by serial dilution into 0.01% DMSO in RPMI 1640 medium or in DMEM. All other chemicals and reagents were of analytical grade.

### Cell culture and stable transfection

Human hepatocarcinoma cell line, HepG2 cell, Huh-7 cell, obtained from the Cell Bank of the Chinese Academy of Sciences (Shanghai, China), were cultured in RPMI 1640 medium or in DMEM supplemented with 100 mL/L fetal bovine serum at 37°C in 5% CO2. When the cell fusion rate reached 80%, in the presence of the liposome Lipofectamin2000 according to the manufacturer’s instructions, HepG2 cells and Huh-7 cells were transfected with plasmid pcDNA3.1-X, which contains the full length HBX sequence, was constructed in mammalian expression vector pcDNA3.1 (Invitrogen, Carlsbad, CA, USA) as described previously[45]. Forty-eight hours post-transfection, the transfected cells were incubated in selection medium containing 800 mg/ml G418. Stable cell lines, named HepG2-HBX and Huh-7-HBX cells respectively, were selected after formation of resistant clones.

### RT-PCR analysis

The total RNAs of HepG2-HBX, Huh-7-HBX and control cells were prepared with Trizol reagent by manufacturer’s instructions. The reverse transcription was performed with TIANScript RT Kit. Primer sequences used for HBX were TGTGAAGCTTATGGCTGCTAGGC and TGTGGAATTCTTAGGCAGAGGTG. Primers for LASP-1 were GTGTATCCCACGGAGAAGGT and TGCCA CTACGCTGAAACCT. Primers for β-actin were GGCATCGTGATGGACTCCG and GCTGGAAGGTGGACAGCGA. The amplification condition was 94°C for 45 s, 58°C (between 55°C to 60°C) for 35 s, 72°C for 1 min for the 35 cycles and a final extension at 72°C for 5 min each. The PCR products were subjected to electrophoresis in 1% agarose gel and visualized by ethidium bromide staining.

### Western blot analysis

For protein extracts, cells were lysed in cell lysis buffer [20 mM Tris PH7.5, 150 mM NaCl, 1% TritonX-100, 2.5 mM sodium pyrophosphate, 1 mM EDTA, 1%Na3VO4, 0.5 μg/ml leupeptin and other phosphatase inhibitors, 1 mM phenylmethanesulfonyl fluoride (PMSF)]. The lysates were collected by scraping from the plates, and then centrifuged at 10,000 × g at 4°C for 5 min. The protein concentration was measured by BCA Protein Assay Kit and adjusted at equal pace. 60 μg total protein was subjected to SDS-PAGE and transferred onto PVDF membranes, the membranes were blocked with 3% BSA in TBS containing 0.01% Tween-20 for 3 h at room temperature, and then incubated with specific primary antibodies: mouse monoclonal anti-HBx antibody (1:500), mouse monoclonal anti-LASP-1 antibody (1:2000), rabbit polyclonal anti-phospho-Akt (1:500), goat polyclonal anti-Akt antibody (1:500) and mouse monoclonal anti-GAPDH antibody (1:3000), respectively, overnight at 4°C. Then, the membranes were incubated with goat anti-mouse IgG-HRP (1:2000), goat anti-rabbit IgG-HRP (1:2000), rabbit anti-goat IgG-HRP (1:2000), separately, for 3 h at room temperature. The protein bands were detected with Immobilon Western Chemiluminescent HRP Substrate.

### Immunofluorescence analysis

The cells were seeded on coverslips at a density of 1 × 10^5^ cells/coverslip and incubated for 24 h. Then the coverslips were washed with PBS, fixed with ice-cold acetone for 10 min. After blocked with 3% bovine serum albumin in PBS for 30 min. the coverslips were incubated with mouse anti-LASP-1 (1:200) at room temperature for 2 h and washed three times with PBS for 10 min/wash. Incubation followed for 30 min at room temperature with FITC-conjugated secondary antibody (1:50). Nuclei were stained using DAPI for 10 min. Then the coverslips were washed with PBS for 10 min/wash. Micrographs were acquired by using an Olympus BX51 fluorescence microscope at × 400 magnification.

### siRNA preparation and transfection

Expression of human LASP-1 was knocked down with siRNA targeting the sequence CACGCCCGAGCTCCAGAGAAT. The negative control siRNA targeting an unknown mRNA sequence GTCTCCACGCGCAGTACATTT was used as a control. Both siRNAs were synthesized by Invitrogen using pcDNA™6.2-GW/EmGFP-miR vector. A blast search against the complete human genome verified that the selected sequences were specific for the target gene. Transient transfection of siRNA was also mediated by liposome Lipofectamine 2000 reagents and carried out as described in the manufacturer’s instructions.

### Cell viability assay

The cells were prepared at a concentration of 3 × 10^4^ cells/ml, and 100 μl of cell suspension were placed into 96-well plates (5 replica wells for each cell line). After incubation for 1, 2, 3 and 4 days, the number of viable cells was analyzed using the Cell Counting Kit-8 (CCK-8) following the manufacturer’s instructions. The optical absorbance at wavelength of 450 nm was measured in a plate reader (ClinBio-128, SLT, Austria).

### Plate clone formation assay

About 200 cells were added to each well of a 6-well culture plate, and each group contained three wells. After incubation at 37°C for 14 days, the cells were washed twice with PBS and stained with Crystal Violet Staining Solution. The number of colonies containing 50 cells was counted under a microscope and the plate clone formation efficiency was calculated using the formula: plate clone formation efficiency = (number of colonies/ number of cells inoculated) × 100%.

### Flow cytometry analysis

After transfection with LASP-1 siRNA for 48 h, 4 × 10^5^ cells were collected, washed twice with PBS and incubated with 1 ml of 75% cold alcohol at 4°C overnight. After washed three times with PBS, the cells were stained with 0.5 ml propidium iodide (PI) solution (BectonDickinson, USA) for 30 min at 37°C in the darkroom. The cell cycle distributions were then measured flow cytometers (FACSCalibur, BectonDickinson, USA), and the results were analyzed by MODFIT 3.0 software (BectonDickinson, USA).

### Transwell assay

For transwell migration assay, 6 × 10^4^ cells in serum-free medium were seeded in each cell culture insert, which contains a polyethylene terephthalate membrane (6.5 mm in diameter, 8 μm pore size). The bottom chamber was prepared with 10% FBS as a chemoattractant. Cells were incubated at 37°C for 24 h. Nonmigrated cells were scraped off the upper surface of the membrane with a cotton swab. Migrated cells were fixed by 4% paraformaldehyde and stained with crystal violet Staining Solution for photography. For quantification, the cells were counted under a microscope at × 400 magnification in five randomly selected fields.

### Wound healing assay

For wound healing assay, the cells were seeded at 2.0 × 10^5^ cells/well in 24-well plates and allowed to reach 100% confluence. A scratch wound was created on the cell surface using a micropipette tip. The wound area was photographed by bright-field microscopy at × 100 magnification at different time points after wounding. The width of the wound was measured and the migration distance was calculated as the formula: migration distance = (wound width at the beginning-wound width after treatment) / 2 (μm). Three separate visual fields were measured in each experiment.

### Statistical analysis

All experiments were performed three times. Semiquantitative analysis of the bands was measured with the Image J analysis software (Version 1.30v, Wayne Rasband, NIH, USA). The data were presented in the mean ± SD format and analyzed by independent-Samples *T* Test or one-way ANOVA (SPSS version13.0), P < 0.05 was considered statistically significant.

## Abbreviations

LASP-1, LIM and SH3 domain protein 1; HBV, Hepatitis B virus; HBx, HBV X protein; HCC, Hepatocellular carcinoma; RT-PCR, transcription polymerase chain reaction; siRNA, Small interference RNA; GAPDH, Glyceraldehyde 3-phosphate dehydrogenase; PI3-K, Phosphatidylinositol 3-kinase; VEGF, Vascular endothelial growth factor; MMP, Matrix metalloproteinase.

## Competing interests

The authors declare that they have no competing interests.

## Authors' contributions

RT and FK contributed equally to this work, designed the study, analyzed the data and wrote the manuscript. RT, LH and HY performed lab work, LH, HY, PZ and DW checked the revised manuscript thoroughly and confirmed all the data given in manuscript. KZ was the project leader who participated in the study design, monitored the work and reviewed the manuscript. All authors read and approved the final manuscript.
