# Correction to: Role of hepatitis B virus X protein in regulating LIM and SH3 protein 1 (LASP-1) expression to mediate proliferation and migration of hepatoma cells

**DOI:** 10.1186/s12985-020-01397-9

**Published:** 2020-08-31

**Authors:** Renxian Tang, Fanyun Kong, Lina Hu, Hongjuan You, Peng Zhang, Weidong Du, Kuiyang Zheng

**Affiliations:** 1 Department of Pathogenic biology and Laboratory of Infection and Immunology, Xuzhou Medical College, 84 West Huaihai Road, Xuzhou, Jiangsu Province, 221002 China; 2 Sektion Experimentelle Anaesthesiologie, Universitaetsklinikum Ulm, 89075 Ulm, Germany

**Correction to: Virol J 9, 163 (2012)**

**http://www.virologyj.com/content/9/1/163**

Following publication of the original article [1], the authors identified an error in Fig. 8, and two errors in Fig. 9a.

In Fig. 8, the western blot results of GAPDH in HepG2 cell groups were mistakenly duplicated from the parts of GAPDH blots in HepG2 cell groups as shown in Figure 5b. During the western blot experiment, we detected the GAPDH expression in HepG2 cell groups and Huh7 cell groups together in the same gel. When organizing the results to prepare Fig. 8, we made a mistake with the GAPDH protein bands associated with HepG2 cell groups and erroneously cut and used the parts of GAPDH protein bands in HepG2 cell groups as shown in Figure 5b.

**Fig. 8:**
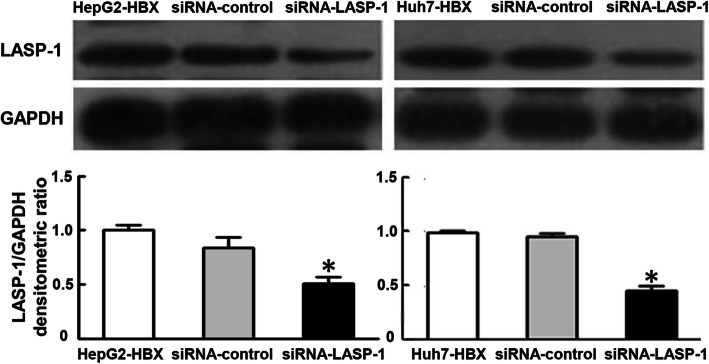
Effects of siRNA againsting LASP-1 on LASP-1 expression in HepG2-HBX and Huh-7-HBX cells. Forty-eight hours after transfection, LASP-1 protein expression was determined by western blot analysis. HepG2-HBX group, Huh-7-HBX group and siRNA-control group were used as the negative controls. *P < 0.05 compared with negative controls

In Fig. 9a, the transwell result in HepG2-Mock group was accidently reused in siRNA-LASP-1 group (HepG2). The transwell result in HepG2-HBX group was incorrectly reused in Huh7-Mock group. The transwell experiments associated with HepG2 and Huh7 cell groups were completed at the same time, and all images related to HepG2 and Huh7 cell groups were stored in the same folder. We found that some images, which contained the overlapping areas with other images, were labeled incorrectly, and caused the errors when we choose and cut the images for preparing Fig. 9a as shown in the article.

**Fig. 9:**
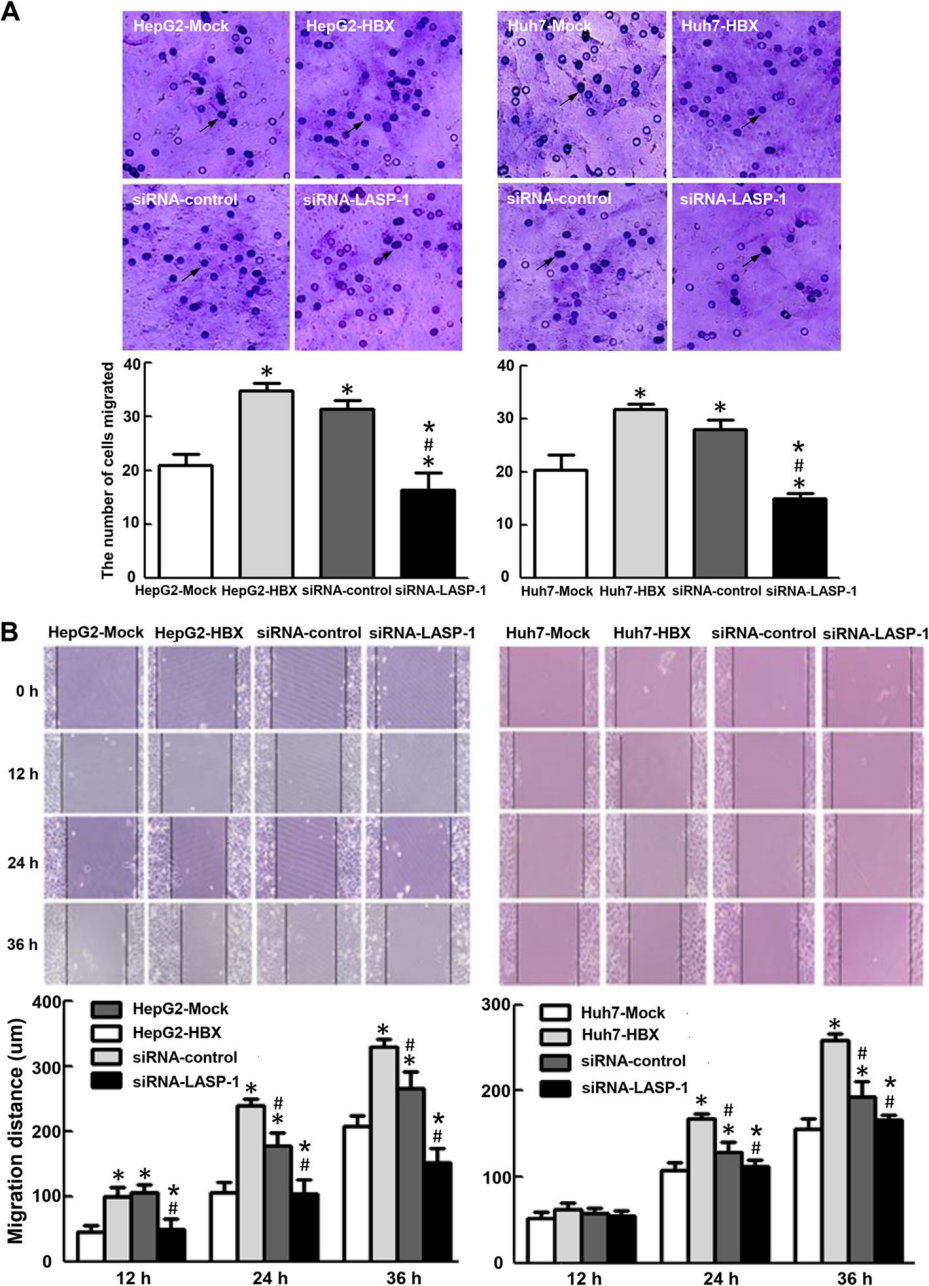
Incearsed LASP-1 mediated by HBx promotes migration ability of HepG2 and Huh-7 cells. The groups were described in Figure 6. **a**. After transfection with siRNA targeting LASP-1 and incubation for 24 h, the cell migration was examined by transwell assay. **b**. HepG2-HBX and Huh-7-HBX cells were treated with siRNA targeting LASP-1 for 24 h, cell migration ability was examined by wound healing assay. The average migration distances of the wound edge in three independent experiments are quantitated below the photograph data. *P < 0.05 compared with control cells. #*P* < 0.05 compared with stable HBx-expressing cells. *P < 0.05 compared with stable HBx-expressing cells transfected with siRNA negative control plasmids

In addition, we also repeated the experiments associated with Fig. 8 and Fig. 9a. We found that the reproducible results were consistent with the original results used in the article, and did not alter our findings.

We declare that the correction does not change the results or conclusions of this paper.
